# *Mycobacterium tuberculosis* exploits the formation of new blood vessels for its dissemination

**DOI:** 10.1038/srep33162

**Published:** 2016-09-12

**Authors:** Helena Polena, Frédéric Boudou, Sylvain Tilleul, Nicolas Dubois-Colas, Cécile Lecointe, Niaina Rakotosamimanana, Mattia Pelizzola, Soa Fy Andriamandimby, Vaomalala Raharimanga, Patricia Charles, Jean-Louis Herrmann, Paola Ricciardi-Castagnoli, Voahangy Rasolofo, Brigitte Gicquel, Ludovic Tailleux

**Affiliations:** 1 Institut Pasteur, Unité de Génétique Mycobactérienne, Paris, France; 2 Univ. Paris Diderot, Sorbonne Paris Cité, Cellule Pasteur, rue du Dr. Roux, F-75015 Paris, France; 3 Institut Pasteur de Madagascar, Unité des Mycobactéries, Antananarivo, Madagascar; 4 University of Milano-Bicocca, Department of Biotechnology and Bioscience, University of Milano-Bicocca, Piazza della Scienza 2, 20126 Milan, Italy; 5 Institut Pasteur de Madagascar, Unité d’Epidémiologie, Antananarivo, Madagascar; 6 INSERM U1173, UFR Sciences de la Santé Simone Veil, Université Versailles-Saint-Quentin, 78180 Saint-Quentin en Yvelines, France; 7 Service de Microbiologie, Hôpital Raymond Poincaré, Assistance Publique Hôpitaux de Paris, 92380 Garches, France

## Abstract

The mechanisms by which the airborne pathogen *Mycobacterium tuberculosis* spreads within the lung and leaves its primary niche to colonize other organs, thus inducing extrapulmonary forms of tuberculosis (TB) in humans, remains poorly understood. Herein, we used a transcriptomic approach to investigate the host cell gene expression profile in *M. tuberculosis*–infected human macrophages (ΜΦ). We identified 33 genes, encoding proteins involved in angiogenesis, for which the expression was significantly modified during infection, and we show that the potent angiogenic factor VEGF is secreted by *M. tuberculosis*-infected ΜΦ, in an RD1-dependent manner. *In vivo* these factors promote the formation of blood vessels in murine models of the disease. Inhibiting angiogenesis, via VEGF inactivation, abolished mycobacterial spread from the infection site. In accordance with our *in vitro* and *in vivo* results, we show that the level of VEGF in TB patients is elevated and that endothelial progenitor cells are mobilized from the bone marrow. These results strongly strengthen the most recent data suggesting that mycobacteria take advantage of the formation of new blood vessels to disseminate.

Tuberculosis (TB) remains a major public health problem, especially in developing countries, with 1.5 million deaths annually worldwide, and it has been estimated that up to one third of the global population carries latent *M. tuberculosis* infection^1^. TB is an extremely complex disease with a large array of clinical manifestations. It is primarily a pulmonary disease that is initiated when *M. tuberculosis*-containing droplets are inhaled into the pulmonary alveoli. After encountering the bacillus, alveolar ΜΦ invade the subtending epithelial layer and secrete several cytokines and chemokines which allow the recruitment and activation of inflammatory cells^2^. This host response to the bacteria results in the formation of granuloma, a structure consisting of concentric layers of infected ΜΦ, foamy ΜΦ, epithelioid cells and multinucleated giant cells surrounded by a mantle of activated T lymphocytes^2^. At the early stage, the granuloma is highly vascularized. As it matures, the blood vessels become less abundant, a central necrotic core appears - as a result of the dying infected MΦ – while a fibrous collagen cuff and other extracellular matrix components develop at the periphery^2^,^3^,^4^.

Granulomas are observed in both active and latent forms of TB. Thus, the formation of a granuloma is not itself indicative of an effective immune response. Nevertheless, although the bacteria are not cleared, granulomas are generally considered to be host-protective structures, containing the primary infection^5^,^6^. This dogma has been challenged in recent years with studies in zebrafish embryos infected with *M. marinum*, showing that mycobacterial growth is indeed facilitated during early granuloma formation^7^. In addition, ΜΦ infected with virulent mycobacteria, together with the neighboring epithelial cells, promote both the recruitment of new uninfected ΜΦ and the formation of ΜΦ aggregates, which facilitate phagocytosis of infected apoptotic cells and increase the bacterial burden^7^,^8^,^9^. Interestingly, ΜΦ actively participate in early mycobacterial dissemination. *M. marinum*-infected ΜΦ frequently leave the primary established granulomas, and migrate both hematogenously and through tissues to trigger new granuloma formation^8^. The formation of new blood vessels orchestrated by the ΜΦ seems to be important for the dissemination of *M. marinum*^10^. Indeed, pharmacological inhibition of the VEGF receptor reduces the infection burden and limits bacterial spreading^10^.

The ability of *M. tuberculosis* to disseminate via the bloodstream and the lymph is well established. As a consequence, TB has been described in virtually all tissues or organs^11^,^12^. Extrapulmonary TB represents about 20% of all TB cases in immuno-competent patients and over 50% of the cases in HIV-infected individuals^13^,^14^. Nearly a quarter of children infected in the first year of life will develop tuberculous meningitis, miliary spread, or bone disease within 2 years^15^. Some observations also support the idea that pulmonary TB may result also from a dissemination of *M. tuberculosis*. Among the individuals initially infected with *M. tuberculosis*, only 10% will suffer from the disease with half of these (mainly infants, children, and immuno-compromised individuals) developing active disease (or primary TB) within one year. Adults usually develop a so-called secondary TB (or post-primary TB) as result of a reactivation of a prior infection^16^,^17^. Secondary TB affects mainly the upper part of the lung^16^, whereas primary TB is likely to develop in any part of the organ. This differential localization may be caused by the early hematogenous spread of the bacteria during primary infection^18^.

Despite significant efforts to understand how *M. tuberculosis* colonizes its host, the cellular and molecular mechanisms involved in mycobacterial dissemination in humans remain poorly understood^19^,^20^,^21^. We report here a study of this important process in the physiopathology of TB by investigating the role of ΜΦ in mycobacterial spread. Using a transcriptomic approach, we identified an angiogenic signature in human monocyte-derived ΜΦ infected with *M. tuberculosis*. The expression of 33 genes, encoding proteins involved in the formation of new blood vessels, was significantly modulated during infection. Consistent with this finding, we show that the potent angiogenic factor VEGF is secreted by *M. tuberculosis*-infected ΜΦ, in an RD1-dependent manner. We next assessed the effect of inhibiting angiogenesis in mice engrafted with human *M. tuberculosis*-infected ΜΦ, and in mice infected with *M. tuberculosis* via the respiratory route. In both models, mycobacterial spread from the site of infection was strongly impaired by the presence of angiogenesis inhibitors. In accordance with our *in vitro* and *in vivo* results, we observed increased angiogenesis in patients with TB. The VEGF concentration in serum of TB patients was elevated, confirming previous studies^5^,^6^,^22^,^23^, and the level of circulating endothelial progenitor cells in blood was also increased when compared to healthy donors. Overall, our data support the idea that mycobacteria exploit ΜΦ for dissemination by inducing the formation of new blood vessels.

## Results

### Expression of genes involved in angiogenesis is up-regulated in ΜΦ upon *M. tuberculosis* infection

**Figure 1:**
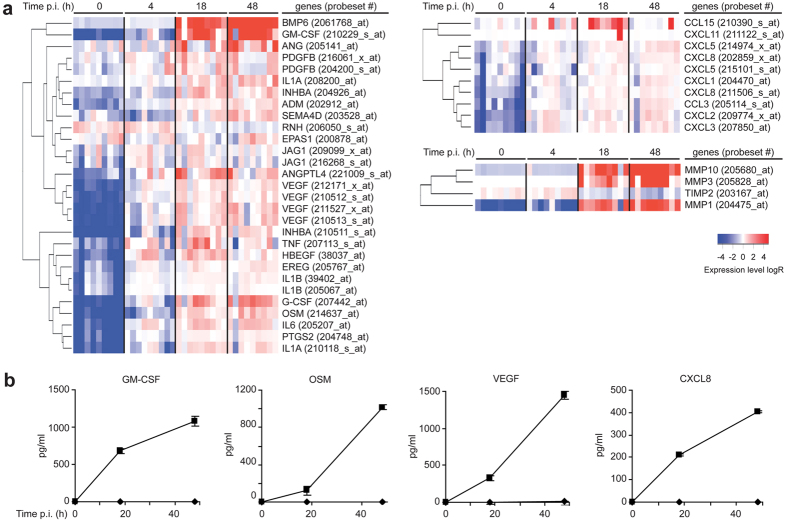
Changes in the expression levels of genes involved in angiogenesis in *M. tuberculosis*-infected ΜΦ. (**a**) Heat-map showing hierarchical clustering according to normalized expression levels of genes that relate to angiogenesis, MMPs, and chemokines. Data are from nine independent experiments and were normalized to determine the log ratio with respect to the median expression of each gene. (**b**) GM-CSF, OSM, VEGF and CXCL8 concentrations were measured by ELISA at various times post-infection (*n* = *3* experiments) in supernatants from uninfected (diamond) and *M. tuberculosis*-infected ΜΦ (square) cultures.

Since the early granuloma is a highly vascularized structure, we hypothesized that angiogenesis might be playing a foremost rule here. Consequently, temporal changes to the transcriptome of human monocyte-derived ΜΦ following *M. tuberculosis* infection were analyzed^24^. mRNAs encoding 31 molecules involved, directly or indirectly, in angiogenesis were strongly up-regulated following *M. tuberculosis* infection (Fig. 1a). The expression of VEGF-A, a key regulator of endothelial cell sprouting and angiogenesis^25^, was 21-fold higher in infected cells than in uninfected ones, suggesting that *M. tuberculosis*-infected ΜΦ promote the formation of blood vessels. Also, the expression of the ribonuclease/angiogenin inhibitor (RNH) gene was down-regulated in *M. tuberculosis*-infected cells. RNH is a powerful inhibitor of angiogenin (ANG)^26^, a major angiogenic factor induced in *M. tuberculosis*-infected ΜΦ (Fig. 1a). Angiogenesis is a stepwise process comprising remodeling of the extracellular matrix (ECM)^27^,^28^ due to several enzymes, of which metalloproteinases (MMPs) are the most representative ones. Amongst the MMPs implicated in angiogenesis^29^,^30^, the gene expression of MMP1, 3, and 10 were up-regulated after *M. tuberculosis* infection, whereas that of TIMP2, an MMP inhibitor, was down-regulated (Fig. 1a). Chemokine encoding genes were also induced (Fig. 1a). Chemokines promote the growth of blood vessels and are involved in the recruitment of circulating endothelial progenitor cells^25^. To validate our transcriptomic data, a selected panel of genes was examined in more detail. We used ELISA to confirm the upregulation of granulocyte-macrophage colony-stimulating factor (GM-CSF), VEGF-A (hereafter VEGF), oncostatin M (OSM), and interleukin 8 (CXCL8) secretion in *M. tuberculosis*-infected ΜΦ (Fig. 1b). The expression patterns of the genes for heparin-binding EGF-like growth factor (HB-EGF), bone morphogenetic protein 6 (BMP6), angiopoietin-like 4 (ANGPTL-4), and inhibin beta A (INHBA) were also validated by testing for the proteins by western blotting (data not shown).

### VEGF secretion correlates with *M. tuberculosis* virulence

**Figure 2:**
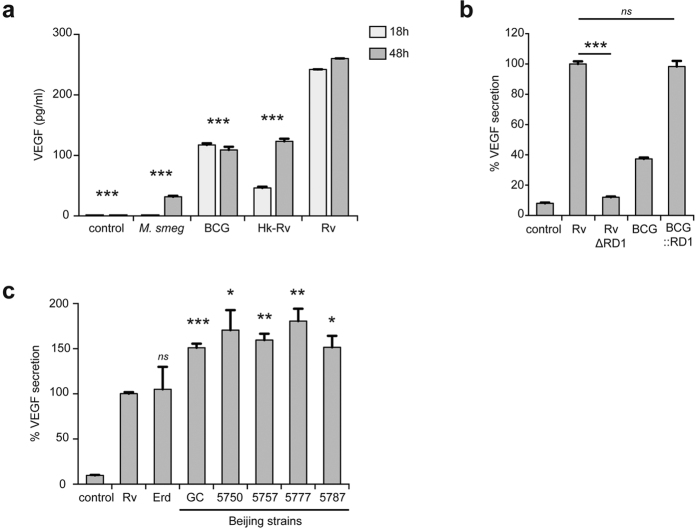
VEGF secretion correlates with virulence of *M. tuberculosis*. (**a**) Human ΜΦ were infected with *M. smegmatis* (*M. smeg*), the avirulent strain BCG, the virulent laboratory strain H37Rv (Rv), or heat-killed H37Rv (Hk-Rv). After 18 and 48 h of infection, VEGF concentrations in the culture supernatants were measured by ELISA (*n* = *3* experiments). (**b**) ΜΦ were infected with H37Rv, H37Rv∆RD1, RD1-complemented BCG (BCG::RD1) and BCG (BCG::pYUB412, control strain harboring the empty cosmid). After 48 h of infection, VEGF concentrations were determined as described previously (*n* = *2* experiments). (**c**) ΜΦ were infected with H37Rv (Rv), Erdman (Erd), and clinical strains of *M. tuberculosis* belonging to the Beijing Family (GC1237, 5750, 5757, 5777 and 5787). Error bars represent the means ± the SEM. *P < 0.05, **P < 0.01, and ***P < 0.001; ns indicates not significant.

We next investigated whether VEGF secretion is restricted to *M. tuberculosis*-infected ΜΦ, or if other mycobacteria species can also induce the release of VEGF. We infected ΜΦ with either *Mycobacterium smegmatis*, which is non-pathogenic; bacillus Calmette-Guérin (BCG), which is an attenuated *Mycobacterium bovis* used for vaccinations; or heat-killed and live *M. tuberculosis* H37Rv. After 48h, ΜΦ infected with *M. smegmatis*, BCG, or heat inactivated *M. tuberculosis* H37Rv secreted 8.2, 2.4, and 2.1-fold less VEGF, respectively, than ΜΦ infected with the virulent strain H37Rv (Fig. 2a). Similar results were obtained 18 h post-infection, although the VEGF concentration was lower in cells infected with *M. smegmatis* or heat-killed *M. tuberculosis*.

We focused on BCG to understand why it is a poor inducer of VEGF. Attenuation of BCG is mainly due to the loss of the RD1 region, which encodes part of the virulent ESX-1 secretion system^31^. Reintroduction of RD1 into BCG results in a significant increase in virulence^32^. Similarly, the phenotype of H37Rv:ΔRD1 is comparable to that of BCG in human macrophages and mice^33^. ESX-1 represents one of the five type VII secretion systems of *M. tuberculosis*, and is responsible for the secretion of two major virulence factors also encoded by RD1, the 6-kDa early secreted antigenic target (ESAT-6) and the 10-kDa culture filtrate protein (CFP-10)^34^. Infection of ΜΦ with RD1-complemented BCG resulted in a similar level VEGF secretion as that observed with *M. tuberculosis* infection (Fig. 2b). Consistent with this observation, ΜΦ infected with RD1 deleted H37Rv secreted 8-fold less VEGF than ΜΦ infected with the parental strain (Fig. 2b). The existence of several *M. tuberculosis* strains, with different virulence potencies, led us to examine the link between VEGF expression by infected ΜΦ and the virulence of *M. tuberculosis* strains. Infection of ΜΦ with highly-virulent clinical strains of *M. tuberculosis*, belonging to the Beijing family, induced an even greater level of VEGF secretion than that observed for ΜΦ infected with the two laboratory strains H37Rv or Erdman (Fig. 2c). These results suggest that VEGF-mediated angiogenesis may be exploited by the bacillus to increase its virulence, or foster its persistence inside the host.

### Human macrophage-controlled angiogenesis allows *Mycobacterium tuberculosis* to disseminate

**Figure 3:**
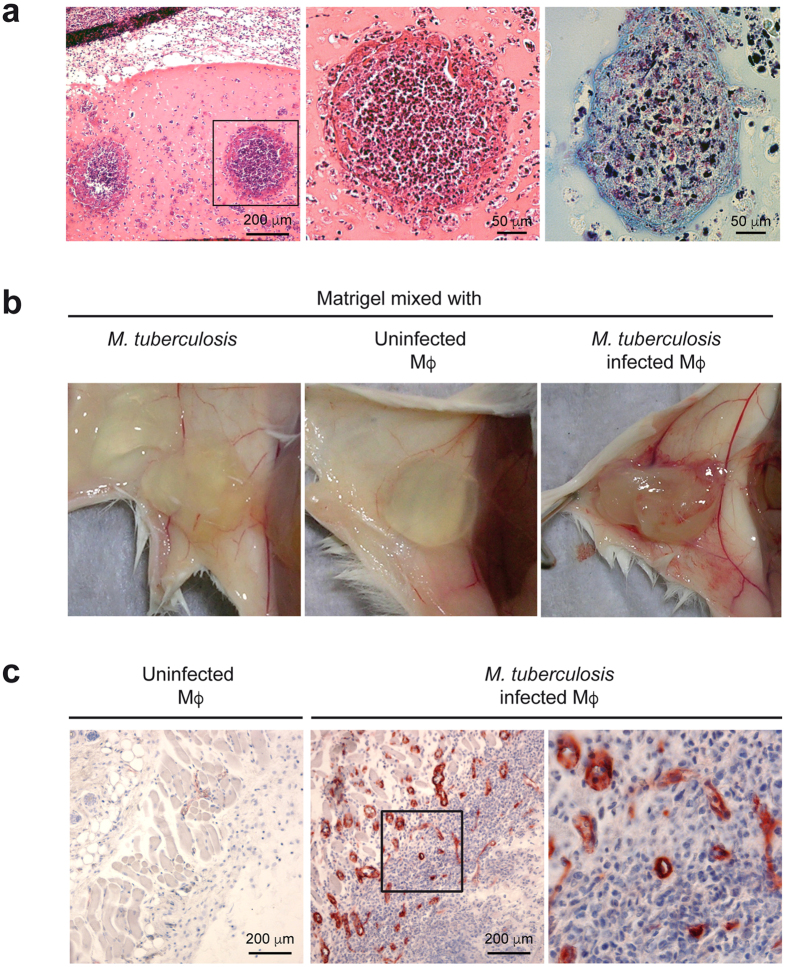
*M. tuberculosis*-infected human ΜΦ induce blood vessel formation. (**a**) Matrigel implants containing uninfected ΜΦ or *M. tuberculosis*-infected ΜΦ were injected subcutaneously into SCID mice. After one week, the Matrigel implants were fixed, sectioned, and stained with hematoxylin and eosin. *M. tuberculosis* was stained using the Ziehl–Neelsen method (right panel). (**b**) Photographs of Matrigels implants after one week. (**c**) Blood vessels associated with Matrigel plugs were stained with an anti-CD31 antibody. Data are representative of three independent experiments (*n* = *3–5* mice/experiment). The regions in squares are shown at a higher magnification in the panels on the right.

Recently it has been shown that *M. marinum* infection triggers angiogenesis in the zebrafish and favors bacterial dissemination^10^. The transcriptomic analysis of *M. tuberculosis*-infected human ΜΦ suggests that these cells also express factors which stimulate endothelial cells to form new blood vessel networks. To test this possibility, we adapted a well-established model used to evaluate the ability of human tumor cells to form metastases^35^. Briefly, Matrigel plugs containing uninfected or infected human ΜΦ were injected subcutaneously into the abdominal area of SCID mice. After one week, well-defined structures which evoke early granulomas, were visible (Fig. 3a). These structures were sites of bacterial multiplication, and some contained a central core of necrotic cells. Of particular interest for our study, the implants with *M. tuberculosis*-infected cells were highly vascularized, whereas unstimulated ΜΦ or bacteria alone were unable to trigger angiogenesis (Fig. 3b). Posterior histological examination of the Matrigel plugs containing infected ΜΦ showed several CD31-positive (CD31) blood vessels, both at the implant periphery and within the gel itself (Fig. 3c).

**Figure 4:**
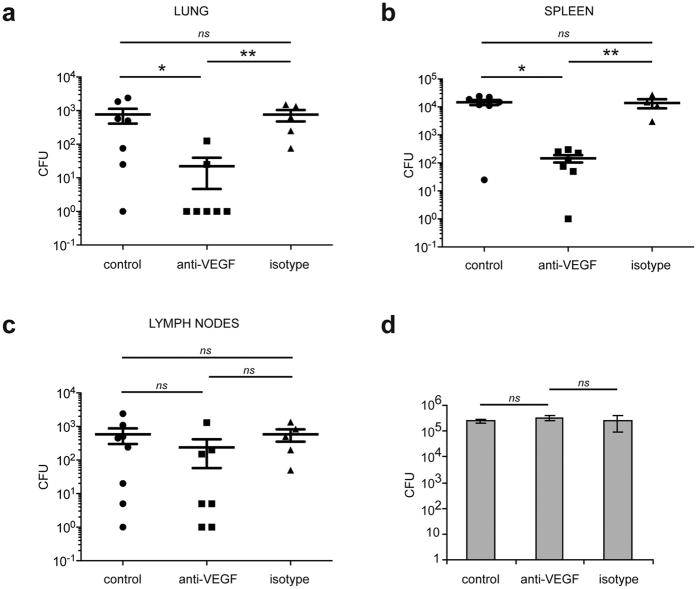
Inhibiting neovascularization abolishes mycobacterial spread without affecting mycobacterial viability. The blocking antibody against VEGF and the corresponding isotype control were mixed in the Matrigel plugs containing *M. tuberculosis*-infected ΜΦ and were injected into SCID mice. Seven days after the implantation, the CFUs were counted in the lung (**a**), in the spleen (**b**), and in the draining lymph nodes (**c**). One representative experiment (out of three) is shown. (**d**) *M. tuberculosis*-infected ΜΦ were incubated *in vitro* with a blocking antibody against VEGF. Cells were lysed 5 days after infection and the CFUs were counted in triplicate (*n* = *2* experiments). Error bars represent the means ± the SEM. *P < 0.05, **P < 0.01, and ***P < 0.001; ns indicates not significant.

VEGF inhibitors are among the best-known anti-angiogenic agents^25^. Having identified VEGF as a putative target molecule for ΜΦ-induced angiogenesis, we assessed the effect of its inhibition using a humanized monoclonal antibody against VEGF. After adding the anti-VEGF antibody into the Matrigel implants, dissemination of the bacillus to the lungs and spleen was markedly hindered – a decrease of about 96% in the lung and 98% in the spleen, Fig. 4a,b). Bacterial spread to the draining lymph nodes was less affected by the anti-VEGF antibody - decrease of 60% though not statistically significant (Fig. 4c). The anti-VEGF antibody had no effect on the intracellular viability of the bacillus (Fig. 4d).

### VEGFR-2 mediates *M. tuberculosis* spread from the lungs

**Figure 5:**
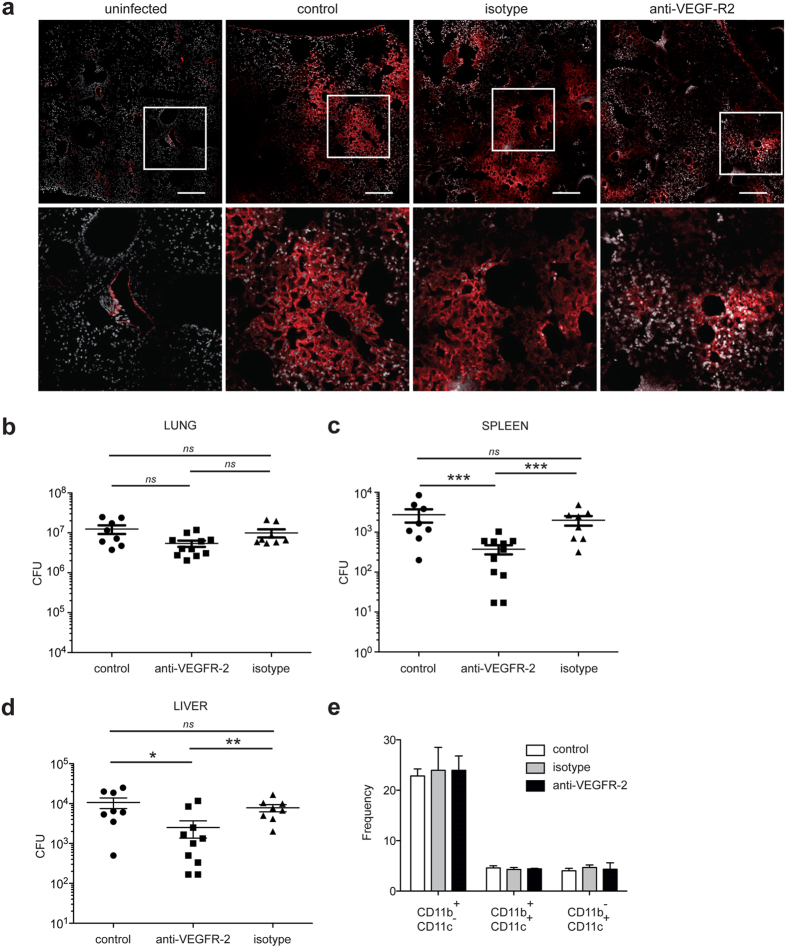
VEGFR-2 dependent signaling allows extrapulmonary *M. tuberculosis* dissemination. Mice were infected intranasally with *M. tuberculosis* and were treated for 14 days with blocking anti-VEGFR-2 antibody or with isotype control. The mice were then sacrificed and (**a**) Blood vessels were visualized using DyLight 594-labeled *L. esculentum* (red). The cells were counterstained with DAPI (white). Scale bars: 200 μm. The regions in the squares are shown at a higher magnification in the lower panels. (**b**) CFUs were counted in the lungs, (**c**) in the spleen, and (**d**) in the liver. One representative experiment out of three is shown. (**e**) Lung myeloid cells were stained with fluorescent anti-CD11b and anti-CD11c antibodies and were analyzed by flow cytometry. The data are representative of two independent experiments. Error bars represent the means ± the SEM. *P < 0.05, **P < 0.01, and ***P < 0.001; ns indicates not significant.

VEGF binds to three receptors: VEGFR-1 and VEGFR-2 which are highly expressed on vascular endothelial cells, and VEGFR-3, for which the expression is confined to the lymphatic endothelium^36^. To confirm that inhibiting angiogenesis abolishes mycobacterial dissemination and to identify which VEGF receptor is involved; the role of VEGFR-1 and VEGFR-2-mediated signaling was evaluated. Mice infected intranasally with *M. tuberculosis* were injected with an anti-VEGFR-1 hexapeptide and an anti-VEGFR-2 neutralizing monoclonal antibody, three times a week, for a two-week period. As expected, *M. tuberculosis* infection induced the formation of new lung blood vessels, and this angiogenic trigger was prevented by injection of the anti-VEGFR-2 antibody (Fig. 5a). Blocking the VEGFR-1 itself had no effect on mycobacterial spread (data not shown). Inhibition of VEGFR-2 mycobacterial growth decreased slightly in the lung (about 57% on average, very close to statistical significance, *P* = *0.052*; Fig. 5b), and strongly impaired mycobacterial spread to the spleen (mean decrease of 86%, Fig. 5c) and liver (mean decrease of 77%, Fig. 5d). It is likely that this effect of the anti-VEGFR-2 antibody is due to its action on endothelial cells. Indeed, repeated VEGFR-2 antibody injection did not affect the percentage or the absolute cell numbers (data not shown) of myeloid cells - namely ΜΦ, dendritic cells and neutrophils - as determined from the expression of CD11b and CD11c (Fig. 5e).

### Angiogenesis occurs *in vivo* in TB patients

**Figure 6:**
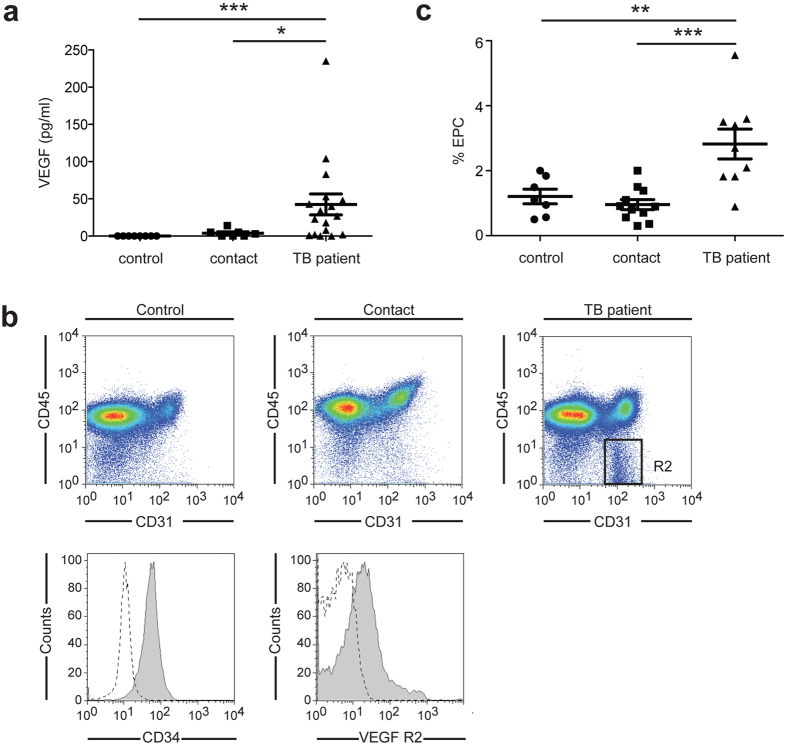
Angiogenesis is an active process in TB patients. (**a**) ELISA testing for VEGF in serum from healthy blood donors (control, *n* = *8*), contacts (*n* = *7*) and TB patients (*n* = *17*). (**b**) PBMC isolated from contacts (*n* = *11*) and from TB patients (*n* = *9*) were stained for CD31 and CD45 (upper two panels). Expression of CD34 and VEGFR-2 was analyzed in cells from R2 (lower two panels). (**c**) Percentage of EPCs for each contact or patient was quantified by flow cytometry. *P < 0.05, **P < 0.01, and ***P < 0.001.

To strengthen evidence that TB induces angiogenesis in humans, we quantified the level of VEGF in TB patients. The presence of VEGF was confirmed by ELISA in the serum of TB patients and it was not detected in the serum of control subjects (Fig. 6a), confirming previous results^5^,^6^,^22^,^23^. Several studies show that circulating endothelial progenitor cells (EPCs) are incorporated into new and preexisting blood vessels during tumor vascularization^25^; therefore, we hypothesized that *M. tuberculosis*-infected ΜΦ facilitate the recruitment and differentiation of EPCs. *M. tuberculosis*-infected ΜΦ express chemokines that may allow mobilization of the EPCs from the bone marrow to the site of infection and the growth factors necessary for their survival and differentiation (Fig. 1a). To test this possibility, we used flow cytometry to evaluate the number of circulating EPCs in the blood of controls, contacts, and TB patients. As shown in Fig. 6b,c, EPCs defined as CD31^+^CD34^+^VEGF-R2^+^CD45^−^ were indeed mobilized from the bone marrow in TB patients.

## Discussion

Infection with *M. tuberculosis* leads to the recruitment of mononuclear cells from neighboring blood vessels and to the formation of highly vascularized granulomas^3^,^4^,^37^. The mechanisms controlling the formation of new blood vessels and the role of neovascularization during TB remain nevertheless poorly understood. Here, we show *in vitro* that human ΜΦ rapidly initiate an angiogenic program upon *M. tuberculosis* infection allowing mycobacterial spread. We identified various genes of which the expression was profoundly modified during infection, and in particular 33 genes encoding growth factors or chemokines promoting recruitment, multiplication, and survival of endothelial cells and EPCs, including VEGF, the most potent angiogenic factor (Fig. 1). *In vivo*, VEGF was detected in the serum of pulmonary TB patients confirming previous studies^5^,^6^,^22^,^23^ (Fig. 6) and we found for the first time that EPCs are mobilized from the bone marrow (Fig. 6).

VEGF is secreted by *M. tuberculosis*-infected human ΜΦ in an RD1 dependent manner. BCG, which lacks the RD1 virulence locus, and RD1 deleted *M. tuberculosis* strains are indeed poor inducers of VEGF relative to *M. tuberculosis* (Fig. 2). Also, *M. tuberculosis* strains belonging to the Beijing/W lineage induced more VEGF secretion than the laboratory strains H37Rv and Erdman. Interestingly, Beijing strains induce significantly less inflammatory cytokines than H37Rv, are particularly virulent in animal models, and are associated with extrapulmonary disease in humans^38^. Other mycobacterial factors might also be involved in the formation of new blood vessels. Injection of beads coated with trehalose 6,6′-Dimycolate (TDM) from BCG into mice causes the formation of vascularized granulomas^39^,^40^. However, in our mouse model, injection of Matrigel mixed with *M. tuberculosis* alone was insufficient to induce neovascularization of the plugs. Further studies are needed to evaluate if TDM is exported from the mycobacterial phagosome *in vivo* and whether this can induce blood vessel formation.

During cancer, angiogenesis promotes tumor progression and metastasis^25^. Angiogenesis may play a similar role during TB and may be implicated in the extrapulmonary forms of the disease. A recent study pleads in favour of this hypothesis. In the zebrafish model, the formation of new blood vessels facilitates *M. marinum* dissemination^10^. Although the basic vascular plan of the zebrafish embryos shows strong similarity to that of other vertebrates^41^, anatomical differences with humans (such as the lack of lungs) and the use of *M. marinum* instead of *M. tuberculosis* make it difficult to extrapolate these results to human TB. However, in our study, we show that inhibiting angiogenesis also impairs *M. tuberculosis* dissemination from the primary site of infection, including the lungs. Inhibiting VEGF signaling using blocking antibodies against VEGF or VEGFR-2 impaired dissemination of *M. tuberculosis* to the lungs, spleen, and liver, and to a lesser extent, to draining lymph nodes (Figs 4 and 5). Colonization of the lymph nodes by the bacillus is likely to involve various mechanisms and to occur via lymph vessels. *M. tuberculosis* may use migrating dendritic cells or ΜΦ as Trojan horses. Indeed, dendritic cells can transport live *M. tuberculosis* from the granuloma to the draining lymph nodes^42^,^43^. Recently, it has been shown that a subset of mycobacteria-infected inflammatory dendritic cells can leave granulomas and form new lesions associated with bacteria-specific T-cells^44^. The formation of new lymph vessels may also be involved. Lymphangiogenesis is regulated by other factors, such as VEGF-C and VEGF-D^45^. VEGF-C expression is up-regulated in mycobacterial granulomas and induces lymph vessel sprouting via VEGFR-3^46^. Lymphangiogenesis thus facilitates the emigration of CD11c^+^ cells out of out of granulomas and into the lymph nodes^46^. Of note, human lymphatic endothelial cells are permissive to *M. tuberculosis* growth in an RD1-dependent manner^47^. Inhibiting VEGF may thus not be sufficient to prevent *M. tuberculosis* dissemination to draining lymph nodes.

Whether *M. tuberculosis* disseminates in humans from the site of infection as free bacteria or within ΜΦ remains to be determined. Using the zebrafish embryo-*Mycobacterium marinum* model of TB, it has been shown that hematogenous dissemination can occur soon after the establishment of the first lesion, within ΜΦ in an RD1-dependent manner^8^. Alveolar ΜΦ are considered to be resident cells but recent work has shown that these cells can migrate following exposure to *Streptococcus pneumonia*^48^. Also, extracellular bacteria released from necrotic ΜΦ may directly invade endothelial cells^49^,^50^ and thereby freely enter the blood. Heparin-binding hemagglutinin adhesion (HBHA), a virulence factor of *M. tuberculosis*, may be involved in these processes. Disruption of the *M. tuberculosis hbha* gene significantly affects mycobacterial interaction with epithelial cells and impairs extrapulmonary dissemination of *M. tuberculosis* in the mouse^51^.

*M. tuberculosis* escape from the granuloma is likely to be a critical factor in disease progression. In adults, most forms of TB occur at a site distant from that of the primary infection and years after the first encounter with the bacillus^16^,^17^. These observations suggest that *M. tuberculosis* in its primary niche is efficiently controlled by the immune system, but defense mechanisms are compromised once the organism gains access to the blood stream. *M. tuberculosis* spread following ΜΦ induced angiogenesis may thus favor mycobacterial latency in immunocompetent adults. Hematogenous dissemination might also allow the bacteria to colonize the thymus and to interfere with T cell differentiation, generating T cells tolerant to *M. tuberculosis*^52^.

Other human pathogens might exploit the blood/lymphatic system and the formation of new vessels. For example, lesions in patients suffering from lepromatous leprosy (which is characterized by formation of highly bacilliferous granulomas) contain numerous CD31-positive micro-vessels not found in patients with paucibacilliary tuberculoid leprosy^53^. Anti-angiogenic agents may be used as adjunctive host-directed therapies in TB as they are for leprosy. VEGF antagonists are already used for the treatment of various diseases and recently, two independent studies have confirmed the feasibility of such an approach. In *M. marinum* infected-zebrafish, the targeting of VEGFR signaling increases the efficacy of the first-line antitubercular drug rifampicin^10^, and in *M. tuberculosis* infected-rabbits, treatment with an anti-VEGF antibody normalizes the vasculature of granulomas, leading to the penetration of small molecules^54^. Angiogenesis is not restricted to the pulmonary form of TB. VEGF levels are also high in patients with tuberculous pleural effusion^6^,^55^ or tuberculous meningitis^56^,^57^. Further studies are needed to evaluate whether individuals who secrete high levels of angiogenic factors upon *M. tuberculosis* infection are more at risk to develop the extrapulmonary forms of the disease.

## Methods

### Ethics Statement

All animal experiments described in the present study were conducted at the Institut Pasteur according to European Union guidelines for handling of laboratory animals (http://ec.europa.eu/environment/chemicals/lab_animals/home_en.htm) and were approved by the Institut Pasteur Animal Care and Use Committee and the Direction Sanitaire et Vétérinaire de Paris under permit #A-75-1600. Sera and PBMC were obtained from healthy subjects (controls), contacts, and TB patients. Written informed consent was provided by all participants. The study was approved by the National Ethics Committee of the Ministry of Health in Madagascar (N°033-SANPF). All methods were performed in accordance with the relevant guidelines and regulations.

### Bacteria, ΜΦ and infection

Human monocytes were purified from buffy coats and differentiated into ΜΦ according to a previously described procedure^58^. *M. tuberculosis* H37Rv was grown from a frozen stock to mid-log phase in 7H9 medium (Becton Dickinson) supplemented with albumin-dextrose-catalase (ADC, Difco). The virulence of bacteria in the frozen stock was checked by infecting C57BL/6 mice intranasally with 10^3^ bacilli: after 21 and 42 days, the bacterial load in the lungs was approximately 10^7^ bacteria. Before infection, bacteria were washed three times and resuspended in 1 ml PBS. Clumps were disassociated by 30 passages through a needle, and then allowed to sediment for 5 min. The density of bacteria in the supernatant was verified by measuring the OD600 and aliquot volumes defined to allow 1 bacterium-per-cell infections: cells were infected in six-well plates with each well containing 2 × 10^6^ cells in 3 ml medium containing Macrophage Colony Stimulating Factor (R&D Systems). After 4 h of incubation at 37 °C, infected cells were washed twice in RPMI 1640 to remove extracellular bacteria and were incubated in fresh medium. In the experiments comparing different mycobacterial strains, ΜΦ were infected with *Mycobacterium smegmatis* mc^2^ 155, BCG Pasteur, or the *M. tuberculosis* strains H37Rv, Erdman, GC1237, 5750, 5757, 5777, or 5787. The bacterial counts were confirmed by counting the colony-forming units (CFUs). Only experiments with similar numbers of bacteria of the different strains were retained for the analyses.

### Enzyme-linked Immunosorbent Assay, ELISA

The concentrations of GM-CSF, CXCL8 (PeproTech), VEGF, and OSM (R&D Systems) in supernatants of uninfected and *M. tuberculosis*-infected ΜΦ and in human serum were determined in duplicate by specific sandwich ELISA as described by the manufacturers. Minimum detection limits were 32 pg/ml (GM-CSF), 16 pg/ml (CXCL8), 15.6 pg/ml (VEGF), and 31.25 pg/ml (OSM).

### *In vivo* Matrigel plug assay

SCID female mice (7–8 weeks old) were purchased from Charles River (France) and maintained according to Institut Pasteur guidelines for laboratory animal husbandry. The *in vivo* Matrigel plug assay was adapted from a protocol from Passaniti *et al*.^59^. Briefly, Matrigel plugs containing 2.5 × 10^6^ untreated human ΜΦ or human ΜΦ infected with *M. tuberculosis* for 18 h, were injected subcutaneously into the abdominal area of anesthetized SCID mice. Anti-VEGF antibody (5 mg/kg; Avastin, Genentech) and isotype controls were included in the Matrigel implants as specified. After one week, the mice were sacrificed. The spleens, lungs, and lymph nodes of each animal were aseptically removed, homogenized, and the bacteria counted as described previously^60^. Matrigel plugs were photographed and prepared for histological examination.

### Determination of bacterial counts

ΜΦ were lysed in cold distilled water with 0.1% Triton X-100. Bacteria were enumerated as previously described^58^ and plated on 7H11. CFUs were scored after three weeks at 37 °C.

### Mouse infection

SCID mice were used to avoid any effects on T cells, as many studies have suggested that VEGF suppresses T cell activation^61^,^62^. Briefly, mice were anesthetized with a cocktail of ketamine (100 mg/kg; Merial) and xylasine (15 mg/kg; Bayer). Mice were infected intranasally with 300 to 500 CFUs of *M. tuberculosis*. To block VEGF/VEGFR-1 and 2 interactions, mice were i.p. injected three times a week with 200 μg/mouse anti-VEGFR-1 hexapeptide (Gly-Asn-Gln-Trp-Phe-Ile, Peptron) and with 25 μg/mouse anti-VEGFR-2 mAb (R&D Systems) as previously described^61^. As negative controls, mice were injected with saline, with reverse peptide (Ile-Phe-Trp-Gln-Asn-Gly, Peptron), or with 25 μg/mouse isotype control Ab (R&D Systems). After 14 days, mice were killed by CO_2_ asphyxiation. Lungs, spleens and livers were harvested, homogenized, and plated on agar for colony counts.

### Immunohistochemistry

Matrigel implants were fixed with zinc for 2 days at 4 °C. After fixation, the tissues were dehydrated in a series of ethyl alcohol concentrations and embedded in paraffin at 37 °C. For histological examination, 5 μm-thick sections were cut and stained with hematoxylin and eosin (H&E) and using the Ziehl–Neelsen method. Vessels were immunostained with an anti-CD31 antibody (Abcam).

### Visualization of the vasculature

To visualize blood vessels, SCID mice were first anesthetized and given intracardiac injections of 100 μg DyLight 594 labeled tomato lectin (Lycopersicon esculentum; Vector Laboratories, Clinisciences). After 5 min, the heart was perfused with PBS-BSA 1% and with 4% paraformaldehyde. Lungs were then frozen and 60 μm-thick sections cut. The slides were mounted with Fluoromount G (SouthernBiotech), and cells counterstained with DAPI to localize the nucleus. A laser-scanning microscope (Zeiss LSM 700) in the tile scan mode was used to capture a mosaic of images.

### Flow cytometry analysis

Cells were isolated from the lungs using a GentleMACS dissociator (Miltenyi Biotec) according to the manufacturer’s protocol. Briefly, lungs were placed in GentleMACS C tubes (Miltenyi Biotec), mixed with DNase I and collagenase D, and homogenized. The fragments were incubated at 37 °C for 30 min under slow mixing and homogenized again. The homogenized lung was passed through a 70-μm pore-size cell strainer, and red blood cells were removed using a red blood cell lysis buffer. Cells were then stained as described previously using the following antibodies: anti-CD11c-PE (eBiosciences) and anti-CD11b-FITC (eBiosciences).

Human PBMC were treated and analyzed as previously described^58^. The following monoclonal antibodies were used: anti-CD31 FITC (BD Pharmingen), anti-CD34 APC (BD Pharmingen), anti-CD45 PE-Cy5 (eBiosciences), and anti-VEGFR2 PE (R&D Systems). Isotype controls were all purchased from BD Biosciences. Fluorescence was analyzed using FACScalibur and CellQuest Pro software (BD Biosciences).

### Patients, sample collection and processing

TB was diagnosed by smear observation and/or bacterial culture and/or from clinical symptoms. All individuals included in the study were HIV negative. 75% and 70% of the contacts and the controls, respectively, were PDD+ positive. Chest X-rays were performed only for contacts with a large PPD reaction. Contacts with abnormal chest X-ray results were considered to be TB patients.

### Statistics

Statistical analyses were performed with GraphPad Prism, using the Mann-Whitney test. A *P* value below 0.05 was considered to be significant.

## Additional Information

**How to cite this article**: Polena, H. *et al*. *Mycobacterium tuberculosis* exploits the formation of new blood vessels for its dissemination. *Sci. Rep.* **6**, 33162; doi: 10.1038/srep33162 (2016).
